# A DNA Barcoding Survey of an Arctic Arthropod Community: Implications for Future Monitoring

**DOI:** 10.3390/insects11010046

**Published:** 2020-01-09

**Authors:** Mikko Pentinsaari, Gergin A. Blagoev, Ian D. Hogg, Valerie Levesque-Beaudin, Kate Perez, Crystal N. Sobel, Bryan Vandenbrink, Alex Borisenko

**Affiliations:** 1 Centre for Biodiversity Genomics, University of Guelph, Guelph, ON N1G 2W1, Canada; gblagoev@uoguelph.ca (G.A.B.); valerie@uoguelph.ca (V.L.-B.); kperez@uoguelph.ca (K.P.); csobel@uoguelph.ca (C.N.S.); aborisen@uoguelph.ca (A.B.); 2 Canadian High Arctic Research Station, Polar Knowledge Canada, 1 Uvajuq Road, Cambridge Bay, NU X0B 0C0, Canada; ian.hogg@polar.gc.ca (I.D.H.); bryan.vandenbrink@polar-polaire.gc.ca (B.V.)

**Keywords:** molecular biodiversity, Insecta, Arachnida, Collembola, Arthropoda, community-based monitoring, tundra

## Abstract

Accurate and cost-effective methods for tracking changes in arthropod communities are needed to develop integrative environmental monitoring programs in the Arctic. To date, even baseline data on their species composition at established ecological monitoring sites are severely lacking. We present the results of a pilot assessment of non-marine arthropod diversity in a middle arctic tundra area near Ikaluktutiak (Cambridge Bay), Victoria Island, Nunavut, undertaken in 2018 using DNA barcodes. A total of 1264 Barcode Index Number (BIN) clusters, used as a proxy for species, were recorded. The efficacy of widely used sampling methods was assessed. Yellow pan traps captured 62% of the entire BIN diversity at the study sites. When complemented with soil and leaf litter sifting, the coverage rose up to 74.6%. Combining community-based data collection with high-throughput DNA barcoding has the potential to overcome many of the logistic, financial, and taxonomic obstacles for large-scale monitoring of the Arctic arthropod fauna.

## 1. Introduction

Climate change is expected to lead to shifts in the phenology and distributions of species [1]. Insects are expected to respond to changing temperatures particularly readily because of their short life cycles, which are heavily influenced by temperature [2]. For example, significant poleward shifts have been reported in the ranges of non-migratory butterflies in Europe [3], and a dramatic change in community structure of parasitic wasps over a period of 50–70 years has been documented in the Canadian subarctic [4]. In the Arctic, changes in the composition of arthropod communities are projected to have a cascade effect on critical ecosystem parameters, such as decomposition, nutrient cycling, and primary productivity [5]. Accurate and efficient arthropod biodiversity monitoring in the Arctic is needed to document changes in community structure and to detect the arrival of new species, including potential disease vectors. Although arthropod communities have been identified as a prospective model for studying general ecological responses to environmental variation, the lack of baseline data has been identified as an important shortfall [6]. Specific responses of different arthropods to climate variation differ considerably, depending on taxon and locality [7]; therefore, detailed knowledge of taxonomy is a necessary prerequisite for tracking the changes happening at the ecological community level. However, the deficient state of taxonomic knowledge of many abundant arthropod taxa and limited availability of expertise in morphological species identification restrict the scale and taxonomic scope of any monitoring schemes based on traditional approaches.

Biodiversity research in the Arctic has typically been conducted by field technicians and taxonomic experts flying in to conduct the work. However, there is a growing movement in biodiversity science toward community-based monitoring (CBM) [8,9] which engages local residents in the research process. CBM incorporates traditional and local knowledge which provides valuable historical context of environmental changes and their impacts on communities over time [10]. A collaborative approach is required to build collegial relationships between community members and scientific researchers, to promote learning, knowledge exchange, and joint use of research results to guide future planning and policy-making [10,11].

In the Western Arctic, CBM has been instrumental in understanding the substantial changes in phenology, height, and relative species abundance of tundra vegetation associated with climate-induced increases in growing season length and active layer depth [8]. Unfortunately, similar monitoring data on arthropods are not yet available. Arthropods have relatively high species diversity even in arctic environments, and the difficulty of species-level morphological identification of most taxa limits the potential of CBM. As a result, arthropod research in the Arctic has so far been conducted by external researchers; and the taxonomic, geographic, and temporal coverage of these studies has generally been limited. A DNA-based approach which bypasses morphological species identifications is not constrained by access to specialized taxonomic expertise and is thus better suited for monitoring arthropods on a large taxonomic scale [12,13]. Furthermore, the involvement of local community members in data collection and interpretation, offers the potential for substantial increase in the spatial and temporal scales and increases societal relevance of such monitoring efforts.

DNA barcoding has become widely used in biodiversity assessment [14,15,16], in studies of community ecology [17,18], and food webs [19,20]. Assessment of species diversity in poorly known and largely undescribed ecological communities is enabled through clustering DNA barcode sequences into molecular operational taxonomic units (MOTU), which can be used as a proxy for species. The Barcode Index Number (BIN) framework [21] implemented in the Barcode of Life Data System (BOLD; http://boldsystems.org/; ref. [22]) is among the most widely used MOTU delimitation approaches. Comparisons between BINs and described species in well-studied arthropod faunas have shown that BINs reconstruct species boundaries with high accuracy [23,24].

The Arctic BIOSCAN project (ARCBIO) is a partnership between the Centre for Biodiversity Genomics at the University of Guelph (CBG), Polar Knowledge Canada (POLAR), and counterpart organizations in the Kitikmeot Region of Nunavut. It aims to assemble a baseline DNA barcode reference library for the region, with a view to developing biodiversity monitoring protocols for the Canadian Arctic that draw from community leadership and employ high-throughput sequencing. Arthropods are among the key focus groups of ARCBIO, which builds on prior surveys, including arthropod community assessments [25], seasonal changes and spatial patterns of spider (Araneae) assemblages [26,27], biodiversity assessment of aquatic insect groups [16], and distribution and temporal changes of dipteran assemblages [28,29].

In this study, we assess the overall arthropod diversity in the vicinity of Ikaluktutiak (Cambridge Bay), Nunavut, using BINs as a proxy for species. We estimate beta-diversity (measured by BIN turnover) among two continuously monitored sites where a standardized sampling protocol was employed. Finally, we compare the relative performance of collecting methods in recovering taxonomically representative samples of arthropod diversity to determine which method or combination of methods would be prospective for CBM purposes, i.e., easy to deploy and low-maintenance, but still capturing a representative majority of arthropod diversity from the study site. We also present a baseline DNA barcode reference library for terrestrial arthropods (insects, arachnids, and springtails) for the Ikaluktutiak area of Victoria Island.

## 2. Materials and Methods

### 2.1. Study Sites

Field work was done from 7 July to 18 August 2018 in the Kitikmeot Region of Nunavut, in the vicinity of Ikaluktutiak (Cambridge Bay, 69.11° N 105.06° W), southern Victoria Island. The area is situated in the Middle Arctic Tundra ecoregion within Bioclimate Subzone D, as defined by the Circumpolar Arctic vegetation map (CAVM) [30]. The dominant plant community type in this area is Nontussock-sedge, dwarf-shrub, moss tundra (CAVM G3) [30]. Several finer-scale eco-sites are represented in the area, defined by winter snow cover and moisture regimes [31]: exposed upland, xeric to mesic habitats are dominated by *Dryas integrifolia*, *Saxifraga oppositifolia*, and *Carex rupestris*; snow-protected, mesic sites are characterized by *Cassiope tetragona*, *Dryas integrifolia*, and *Salix reticulata*; protected lowland sites are dominated by *Salix richardsonii* and *Carex aquatilis*, and mosses. The area is underlain by cobbly bouldery limestone till with granite inclusions; in upland (xeric) habitats, stones occupy up to 90% of the surface area [31].

Because of the goal to assemble a baseline DNA barcode reference library for arthropod diversity in the Ikaluktutiak area, efforts were made to cover different terrestrial eco-site types and freshwater bodies (tundra ponds, streams) opportunistically during general collecting activities. Moreover, two continuous monitoring sites have been established: one in proximity to the Canadian High Arctic Research Station (CHARS) Intensively Monitored Area (IMA site) on the northern shore of Grenier Lake [32] (69.2186° N, 104.9258° W) and the second site near Water Lake (WL site, 69.1323° N, 105.0621° W)—a more disturbed habitat 1 km north of the hamlet of Ikaluktutiak. Following setup, the sites were checked every other day; however, deviations from this schedule had to be made for the less accessible IMA site because of weather and logistical constraints.

### 2.2. Collecting Methods

The following active collecting methods and stationary traps were used (Table 1):

- Malaise traps—three Townes-style Malaise traps (BugDorm, MegaView Science Co., Ltd., Taiwan) were installed at continuous monitoring sites: one near Water Lake and two near IMA. Samples were collected in 500-mL plastic bottles filled with ~350 mL of 95% ethanol, which were exchanged on a weekly basis. (NB: During this study, the polyester support strings used to anchor Malaise traps installed at the CHARS IMA site were damaged by arctic foxes, which, combined with strong wind gusts, caused the traps to collapse completely or partially between service dates. As a result, stock ropes were replaced with eight 1/16 inch galvanized “aircraft cables” (2.5 m in length). Cables were fitted with aluminum oval sleeves to form loops at the ends and a round swivel eye simplex snap was used to attach each cable to the trap. The distal end of each cable was anchored to the ground with a metal peg, reinforced as needed with heavy stones. These modifications prevented further collapsing of the traps for the remainder of the field season).
- Pan traps—ten yellow shallow plastic bowls (9” diameter) were installed at each of the monitoring sites and half-filled with soapy water. Traps were checked every other day, and the two-day catch from each pan trap site was pooled into a single bulk sample. Collected specimens were strained through a Nitex nylon fabric with a mesh size of 50 µm and fixed in 95% ethanol.
- Pitfall traps—a line of 10 translucent wide-mouth 500 mL plastic cups was installed at 3 m intervals at each monitoring site. The traps were half-filled with soapy water and capped with a 10 mm steel mesh to exclude vertebrate by-catch. The checking schedule and specimen collecting procedure was the same as for pan traps.
- Sweep-netting—arthropods were collected from air and from vegetation with a standard insect sweep-net, then hand-sorted or aspirated, followed by placement in a kill jar or fixation with 95% ethanol. At each of the two monitoring sites, sweep-netting was performed weekly for five continuous minutes (six times at WL site and five times at IMA site).
- Soil and litter sifting—three 30-m soil sifting transects were established parallel to the pitfall trap line at each of the two monitoring sites, with the middle transect adjacent to the pitfall traps, and one transect on either side of the pitfall line at a distance of 10 m. Ten ca. 1-L samples of litter and surface soil were taken with a trowel at 3-m intervals, sifted through a 5-mm mesh to remove larger debris, and pooled into a single bulk sample. The resulting soil and litter samples (ca. 3 L in volume) were placed in Berlese funnels for one week to extract specimens, which were collected in vials with 95% ethanol. The soil transects were sampled four times during the study period for each continuous monitoring site. In addition to the transects, samples of varying size were taken opportunistically by hand or with a trowel from soil surface, moss, debris, remains of dry vertebrate carcasses, or leaf litter in various habitats across the study area. Each sample was sifted on site through a 5 mm mesh, followed by hand sorting in the lab and fixation of all extracted organisms in 95% ethanol.
- Aquatic sampling—arthropods were collected from the water column, submerged vegetation, and bottom sediments of tundra ponds and streams using a hand-held plankton net, D-net or small hand net, then strained from water and fixed with 95% ethanol.
- Freehand collecting—individual arthropods were opportunistically picked by hand, with forceps, or hand-netted, followed by fixation with 95% ethanol.

Stationary traps were only used at continuous monitoring sites. Active methods were used opportunistically for general collecting throughout the study area, and in a regimented fashion at long-term monitoring sites, consistent with the Standardized Sampling protocols designed by the CBG’s field teams for monitoring arthropod biodiversity (deWaard et al. in prep.). A total of 450 bulk samples (lots) were obtained as a result of all arthropod collecting efforts.

### 2.3. Sample Processing and DNA Barcode Sequencing

Collection materials were accessioned and processed at the CBG, following the single-specimen barcoding workflows outlined in deWaard et al. [33], with exceptions, as described below. Because the goal of the project was to create a DNA barcode reference library for future monitoring, bulk sample (lot) sorting was prioritized to maximize taxonomic diversity, while reducing the number of replicates per species through initial morphotaxonomic screening. Lots from continuously monitored sites were prioritized over lots from general collecting, except for Malaise trap materials, of which only lots collected in odd-numbered weeks (1, 3, 5, etc.,) were processed in order to reduce the costs of analysis. In total, 428 of the 450 lots were processed, of which 34 were sorted completely; the remainder had specimens remaining after sorting. Morphospecies sorting of each continuous monitoring sample began with picking all specimens representing taxa other than the highly abundant Diptera and Hymenoptera for analysis. A subset of each morphospecies of Diptera and Hymenoptera was then picked for analysis by senior technical staff with extensive experience in arthropod specimen sorting. General collecting samples were screened and morphospecies sorted by taxonomic experts in Araneae and Coleoptera, with a subset of remaining specimens from other groups chosen for analysis. A total of 24,198 arthropod specimens were selected for DNA barcode analysis. They belonged to three classes: Insecta (19,661), Collembola (904), and Arachnida (3633). Among arthropods isolated from aquatic samples, 264 specimens belonged to crustacean taxa. Although submitted for DNA barcoding analysis, they were not part of this study; therefore, they are excluded from the figures reported here.

DNA extraction, PCR amplification of the COI barcode region, and sequence analysis were done at the Canadian Centre for DNA Barcoding (CCDB, Guelph, Ontario, Canada; ccdb.ca)—the Genomics Unit of CBG. Single molecule, real-time sequencing was performed on the SEQUEL platform (Pacific Biosciences, Menlo Park, CA, USA) using the methodology described by Hebert et al. [34].

### 2.4. BINs and Taxonomic Assignment

Taxonomic diversity among the successfully sequenced specimens was assessed by using the built-in BOLD algorithm for assigning DNA barcode sequences to Barcode Index Number clusters (BINs)—molecular operational taxonomic units used as a proxy for species [21]. Using this algorithm, sequences over 300 base pairs in length can be assigned into an existing BIN, but the founding sequence of a new BIN must be at least 500 base pairs long [21]. Further taxonomic assignments were made by checking the sequences against existing taxonomically identified reference data on BOLD, using built-in queries. When a BIN contained records with specimens identified to a concordant family, genus, or species, any unidentified specimens assigned into this BIN received this identification. Specimens representing a BIN new to BOLD were queried using the BOLD identification engine. The new BIN received a family-level identification if it showed <10% sequence divergence from previously identified sequences, and a genus-level identification if the sequence divergence was <5%. In addition, the query sequence had to be clustered within other BINs with the same taxonomic assignment in order to be assigned to that family or genus [33]. Additional reviews were conducted using Neighbor-Joining trees coupled with images of the associated voucher specimens. When possible, representative specimens of some of the new BINs were also identified morphologically by taxonomic experts.

### 2.5. Archiving and Imaging

Specimens were archived in the natural history collection at CBG (BIOUG), although separated from the main archive, so that a synoptic collection could be devised for later deposition at the CHARS campus in Ikaluktutiak. Residual DNA extracts were deposited in the CBG DNA archive for quality control purposes. Specimens and DNA extracts are available for study upon request.

Records that gained BINs were queried within BOLD to determine which BINs lacked photographs. A single representative specimen was photographed from each new BIN. Efforts were made to select a specimen in good condition when possible. Specimens were photographed at high resolution and the images are accessible through both specimen and BIN pages under Creative Commons (BY-NC-CA) license.

### 2.6. Data Analyses

All specimen records and associated provenance data, as well as DNA barcode sequence data, were uploaded to BOLD (http://boldsystems.org) [22]. All analyses were restricted to the three major classes of Arthropoda which formed the vast majority of the processed material: Arachnida, Collembola, and Insecta. All specimens that failed to produce sequences of sufficient length and quality to get assigned to a BIN were excluded from analyses. Any sequences confirmed to be contaminated by non-target DNA were likewise excluded. The final dataset included 18,096 specimens with high-quality sequences, which gained BIN assignments by 31 October 2019.

The completeness of species coverage was estimated with accumulation curves based on the BIN data. With only a subset of each sample processed, multiple accumulation curves were created for different orders to account for the abundance bias and extrapolated to 100 sites with EstimateS 9.1.0 [35]. Samples were grouped into sites (*n* = 53) with distinct GPS coordinates to account for collective effort at one particular locality split among several samples (e.g., multiple jars/vials per collecting event) and for collecting at the same site on different dates. Chao1 estimates were not calculated for insect orders that were represented by less than 10 species (Ephemeroptera, Plecoptera, Trichoptera, Thysanoptera) and for orders of mites and collembolans where some order-level assignments were inconclusive or based solely on molecular data.

Comparative species diversity captured by different trap types and collecting methods and taxonomic overlap between them were estimated based on the presence or absence of BINs in the samples collected with the corresponding technique. The vocabulary used in field notes was standardized to aggregate records, based on the type of activity (i.e., passive or active trapping) and based on the targeted habitats (i.e., aquatic or terrestrial). The traps left unattended for a certain period of time, i.e., Malaise trap, pitfall trap and pan trap, were collectively considered passive terrestrial collecting methods. All aquatic collecting methods were considered active, because no traps targeting aquatic habitats were used. Active terrestrial methods included soil transects and sweep netting at the continuously monitored sites, as well as opportunistic sifting, sweep netting, and free hand collecting outside these sites.

### 2.7. Data Availability

All DNA barcode sequences and associated provenance data are accessible through a public dataset on BOLD (dx.doi.org/10.5883/DS-CHARS). The sequences are also available through NCBI GenBank, accession number range MN665381–MN683476. See Supplementary Material, Table S1 for detailed list of records.

## 3. Results

### 3.1. Overall Arthropod Diversity and Sequencing Success

Of the 24,198 specimens of Insecta, Collembola, and Arachnida selected for molecular analysis, 18,616 (77%) produced sequences of sufficient quality for BIN assignment, and 18,096 (75%) were assigned to BINs before the dataset was finalized on 31 October 2019. Sequencing success varied between taxa, and was comparatively low for mites (Acari), of which only 34% were assigned to BINs. A detailed summary of sequencing success by taxon is provided in Supplementary Material, Table S2. In order to test whether the specimen collecting medium affected the sequencing success, we compared the success rates between Malaise traps (specimens collected directly in 95% ethanol) and pan traps (specimens collected in soapy water over a two-day period and fixed in ethanol when the traps were serviced), which target largely the same set of higher arthropod taxa. Taxon-specific success rates were generally similar between these two methods (see Table S2). For Diptera and Hymenoptera—the two most abundant insect orders in these two trap types—the respective success rates were 82% and 78% for Malaise traps, and 88% and 79% for pan traps. The overall sequencing success rate was 83% for Malaise traps and 82% for pan traps.

The 18,096 records were assigned to 1264 BINs, of which 304 (24%) were new to the BOLD database. The remaining 960 BINs were previously recorded outside Victoria Island. Insects represented the bulk of the dataset, both in the number of specimens (15,902, 87.9%) and BINs (999, 79.0%) (Table 1). The two most abundant and diverse insect orders were Diptera and Hymenoptera, representing 644 (51%) and 255 (20%) BINs, respectively. Species-level identifications were made for 357 (28%) BINs. Of the remaining BINs, 387 (31%) were identified to genus, 79 (6%) to subfamily, and 409 (32%) to family. Twenty BINs, all representing mites, were lacking order-level identification at the time of writing.

**Figure 1:**
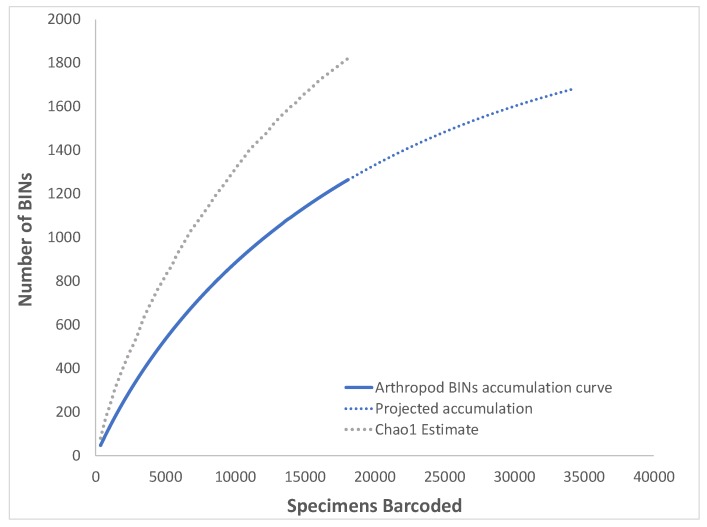
Estimated accumulation curve and overall taxonomic diversity assessment for all arthropods barcoded.

**Figure 2:**
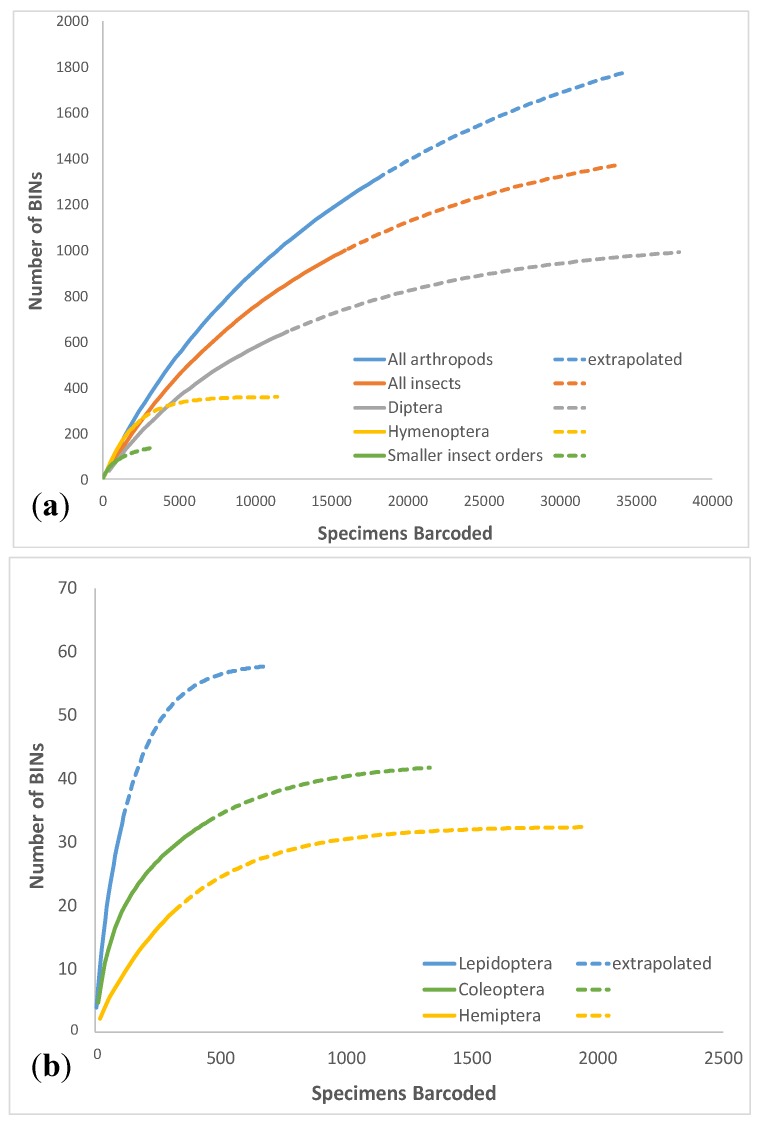

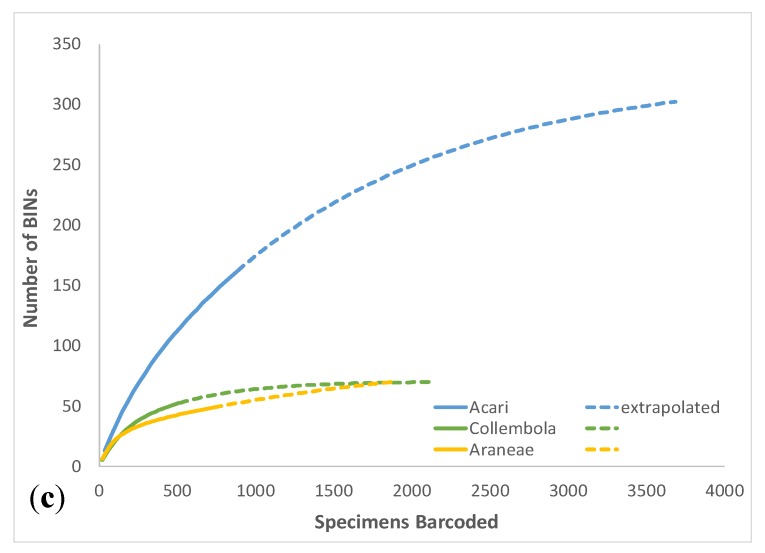
Accumulation curves estimated for different arthropod groups using EstimateS. (**a**) Most abundant insects; (**b**) less abundant insect orders; (**c**) non-insect terrestrial and soil arthropods.

Chao1 estimate for the entire arthropod dataset calculated using EstimateS was 1820 ± 72.9 (lower-upper 95% confidence interval bound estimate 1695–1983), suggesting that DNA barcodes were recovered from approximately 69% of species potentially occurring in the study area, although the Chao1 estimate trend line did not reach an asymptote (Figure 1). This ratio was consistent with that calculated for most lower-level taxa (Table 2) which varied between 67–80%. The two exceptions were Collembola and Hemiptera, where recovered BINs represented 29% and 43% of the estimated diversity, respectively. These taxa also had the highest discrepancies between lower and upper 95% confidence interval bound for Chao1 estimates: 10.7 and 5.9-fold, compared to 1.2–2.5-fold for the remaining arthropods (Table 2). The extrapolated accumulation curves (Figure 2a,b) were generally in agreement with the Chao1 estimators calculated for the various taxa, except for Collembola, for which the extrapolated diversity was ca. 60 BINs (Figure 2c) but the Chao1 estimator was 180.8 (lower-upper 95% confidence interval bound estimate 74.8–802.1).

### 3.2. Diversity at Continuously Monitored Sites

**Figure 3:**
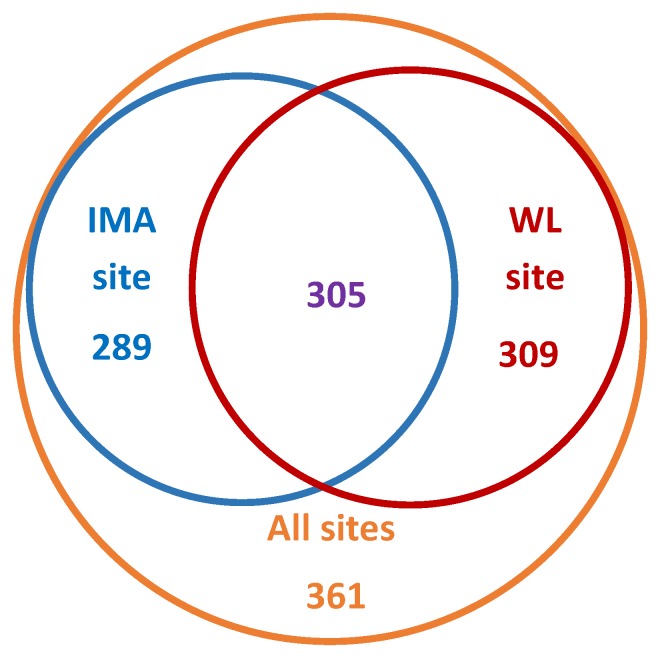
Venn diagram showing the number of Barcode Index Numbers (BINs) collected at each of the two continuously monitored sites, compared against the overall diversity observed. IMA—CHARS Intensively Monitored Area north of Grenier Lake; WL—Water Lake north of Cambridge Bay.

Observed BIN diversity at the two continuously monitored sites (Water Lake and CHARS IMA) was roughly equal; 594 and 614, respectively, with about one-half of BINs shared between them. Together, specimens collected at the two sites accounted for 71% of the overall diversity found in the Ikaluktutiak area of Victoria Island (Figure 3).

### 3.3. Comparative Performance of Sampling Methods

**Figure 4:**
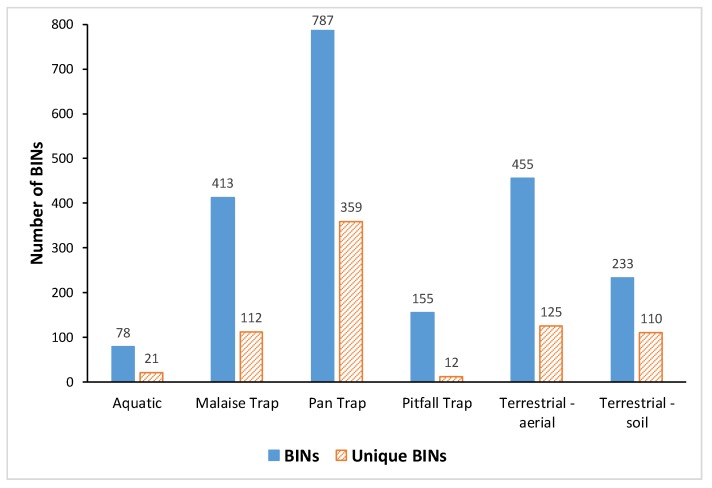
Observed species richness (BINs) and number of unique BINs sampled by the main categories of collecting protocols.

**Figure 5:**
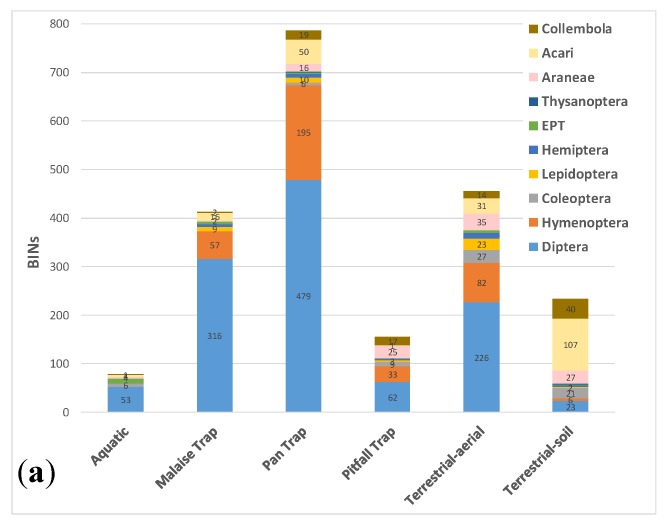

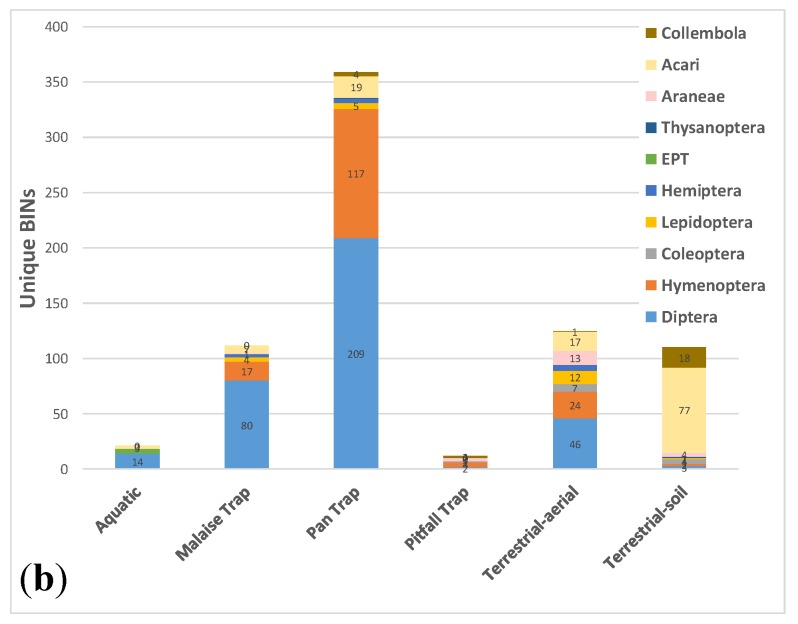
Species richness of major taxonomic groupings of arthropods sampled using different collecting methods: (**a**) all BINs collected by each method; (**b**) unique BINs collected by each method. (EPT—Ephemeroptera, Plecoptera, and Trichoptera combined).

**Figure 6:**
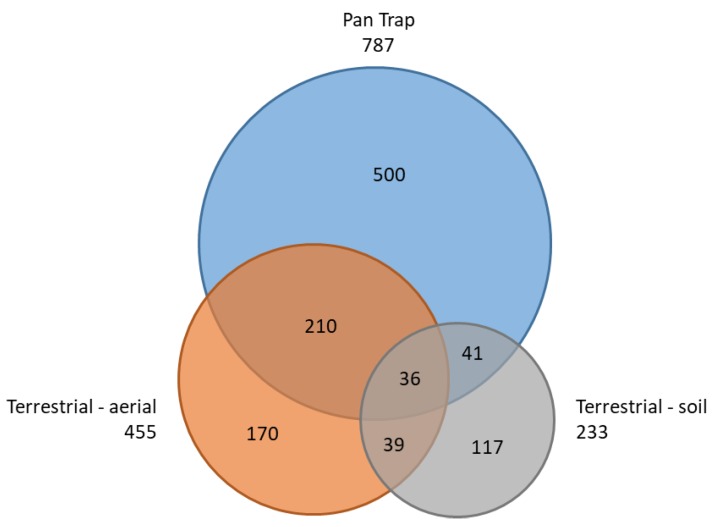
Venn diagram of all BINs collected among pan traps and both terrestrial active methods.

Pan traps captured both the highest number of BINs overall (787, or 62% of the total BIN count) and the highest number of unique BINs (359) not caught with any other sampling method (Figure 4). The next highest BIN count was recorded by terrestrial-aerial active sampling (sweep-net and freehand; 455 BINs, of which 125 unique), followed by Malaise traps (413 BINs, of which 112 unique). Table 3 provides a summary of the BIN counts and overlap between the sampling methods. The taxonomic composition at the order level was similar between pan traps and malaise traps, with Diptera and Hymenoptera overwhelmingly dominating the catch (Figure 5). Active terrestrial sampling methods, especially soil and leaf litter sifting, captured a distinctly different set of higher taxa (Figure 5). Figure 6 shows the BIN overlap between pan traps, sweep-netting (terrestrial–aerial), and soil and litter sifting (terrestrial–soil), which collectively accounted for 88% of the BINs recorded.

## 4. Discussion

### 4.1. Arthropod Diversity at Ikaluktutiak (Cambridge Bay)

In his remarkably comprehensive synthesis of the arctic Arthropoda known at the time, Danks [36] estimated that a minimum of 2104 non-marine arthropod species (excluding Crustacea) had been recorded from arctic North America by the late 1970s and that this number likely covers no more than half of the actual fauna. This study recorded 1264 BINs during a single field season in a restricted area in the Middle Arctic ecoregion (within 50 km of Cambridge Bay) that is geobotanically uniform (Nontussock-sedge, dwarf-shrub, moss tundra [30]). Thus, our observed BIN count for the Ikaluktutiak area alone is equivalent to 60% of the figure provided by Danks; or 87%, if corrected for the mean Chao1 species richness estimator. Although additional species records and new species descriptions have been published since Danks’s work, alpha-taxonomic diversity of Arctic arthropods remains insufficiently known. It should be noted that 24% of the BINs recorded in this study were new to BOLD, which currently contains approximately 660 thousand BINs globally, with the majority of them being arthropods. This is a clear indication that DNA barcode coverage of Arctic arthropod diversity is likewise far from complete. Within the Ikaluktutiak area, future surveys in currently under-sampled habitat types, for example, the sandy Long Point area west of the hamlet, are also likely to increase both the observed and estimated species richness for the area. Additional collecting in aquatic habitats is also required to provide a more comprehensive baseline for arthropod biodiversity.

### 4.2. Comparative Assessment of Collecting Methods

**Figure 7:**
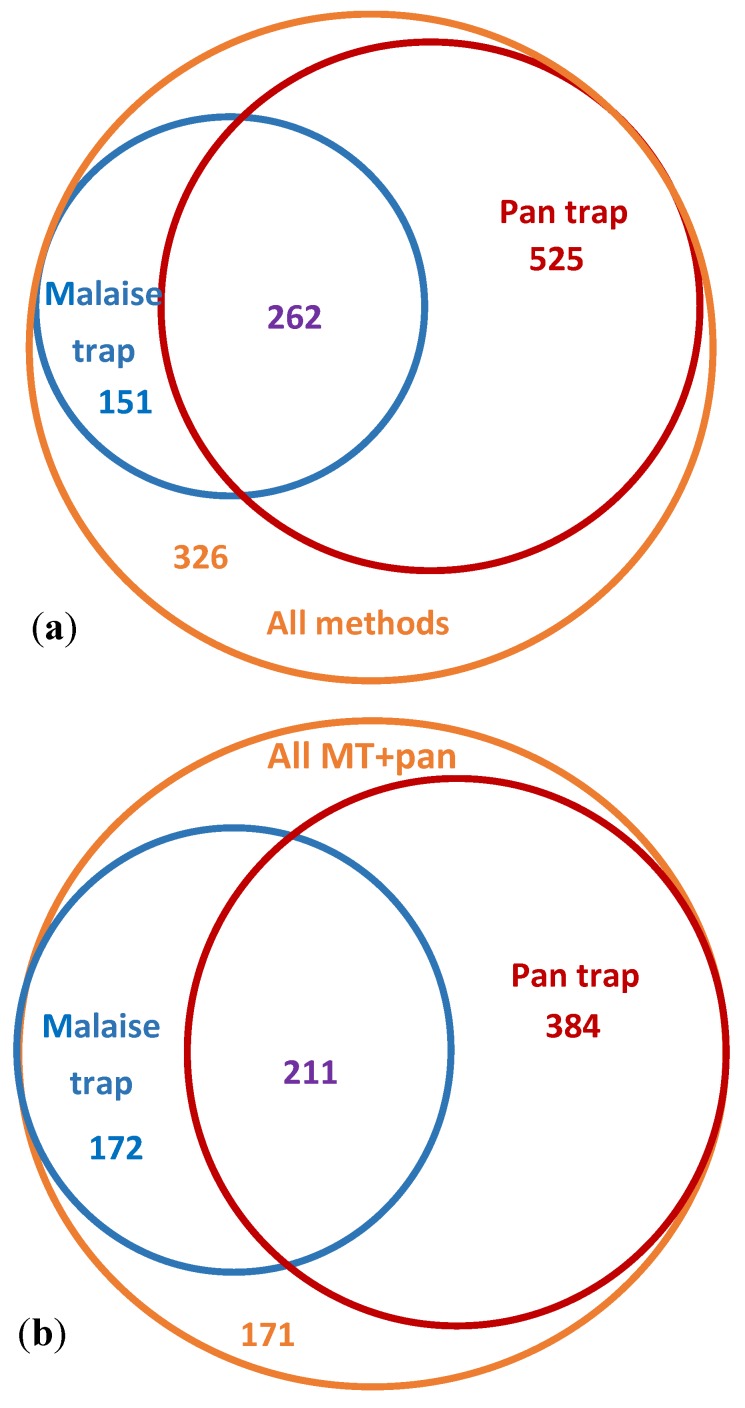
Comparative performance of Malaise traps and pan traps in the summer of 2018, corrected for phenology. (**a**) All Malaise trap samples compared against all pan trap samples and all other collecting methods. (**b**) Malaise and pan trap sampling from approximately similar date ranges compared against all BINs from Malaise and pan traps combined. Dates of Malaise trap sampling: Water Lake site—12–19 July, 24–30 July, 6–13 August; CHARS IMA site—25–31 July, 8–15 August. Dates of pan trap sampling included: Water Lake site—10–17 July, 24–30 July, 5–13 August; CHARS IMA site—25–31 July, 8–15 August.

Among the collecting methods used in this study, yellow pan traps captured the largest number of BINs overall and by far the highest count of unique BINs (i.e., not recorded with any other method). In particular, pan traps sampled a greater diversity of arthropods, compared to Malaise traps (Figure 5). This could be explained in part by possible phenological bias—a result of analyzing all pan trap samples, but only every other week of Malaise trap samples, as per the standard Malaise trapping protocol used at the CBG [33]. However, even after accounting for this bias by removing every other week of pan trap sampling (Figure 7), pan traps demonstrated a clear advantage.

Malaise traps are widely used in surveys of flying insect diversity and are particularly effective in surveying megadiverse taxa such as Hymenoptera and Diptera e.g., [33], but their suboptimal performance in arctic environments was noted earlier by Danks [36]. Field deployment of Malaise traps in the Ikaluktutiak area presented additional logistical challenges, as a result of their initial damage and collapsing caused by arctic foxes and strong wind gusts (see Methods section). This may have contributed in part to their reduced performance and needs to be considered when planning arthropod sampling in the tundra.

The different types of collecting methods used in this study are geared toward specific life forms of arthropods, thereby preferentially sampling certain taxa and life stages, while leaving part of the fauna poorly represented or completely unsampled. Pan traps were effective at sampling Diptera and Hymenoptera, which represent the vast majority of the species-level diversity in the Ikaluktutiak area (71% of observed BINs); thus they also sampled the majority of alpha-taxonomic diversity overall (787 BINs, 62%). Sweep-netting captured the second highest number of total and unique BINs, and, combined with pan traps, accounted for 84% of the overall diversity. We expect that the taxonomic overlap between pan trap, Malaise trap, and sweep-netting samples would increase with additional sampling effort. These methods are biased toward flying and plant-dwelling insect groups, particularly Diptera and Hymenoptera, leaving the flightless and ground-dwelling taxa poorly represented. As expected, these methods were inadequate in surveying arthropods inhabiting soil and leaf litter, such as the majority of arctic species of Acari and Coleoptera.

Pitfall traps are a standard method for sampling ground-dwelling arthropod species [37], but the diversity sampled with pitfall traps in this study was surprisingly low—155 BINs in total, of which only 12 were not captured by any other method. By contrast, soil and leaf litter sifting produced most of the unique BINs of Acari and Collembola (77 and 18 BINs, respectively), and the total number of unique BINs recorded with sifting was on par with Malaise traps (110 vs. 112 BINs; Figure 4 and Figure 5). Because the sequencing success for Acari and Collembola was comparatively low (only 34.1% of Acari and 58.4% of Collembola received BIN assignments; see Supplementary Material, Table S2), the diversity of these taxa is apparently underestimated in this study, and the true diversity captured by soil and litter sifting was higher than recorded here.

The combined effort of pan traps and sifting produced 943 BINs (74.6%), and adding sweep-netting samples to these two methods raises the coverage to 1113 BINs (88%; Figure 6). This suggests that an increased effort of pan trapping combined with soil sifting and sweep-netting is likely to provide sufficient taxonomic representation for future monitoring activities in the middle arctic tundra.

### 4.3. Implications for Community-Based Monitoring

From a CBM perspective, the efficient performance of pan traps is an encouraging result, because they are relatively cheap, lightweight, and easily deployed/serviced. Their ease of use and high portability also makes them an excellent method for opportunistic, short surveys of Arctic insect diversity that can be conducted in parallel with other field work activities, e.g., during day trips to remote locations. Furthermore, comparison of DNA barcode sequence recovery from pan traps vs. Malaise traps (Table S2) did not show any adverse effect of using soapy water as the interim collecting medium. This obviates some of the logistical complications of using ethanol in trap field deployment; however, transfer into ethanol during trap servicing or at the base camp, followed by storage at low temperatures is imperative for long-term DNA preservation.

Because a comprehensive monitoring program should cover ground-dwelling, as well as volant taxa, pan traps need to be supplemented with other sampling methods (e.g., soil sifting) whenever logistically feasible. In this study, soil and leaf litter sifting proved to be more efficient than the widely used pitfall traps in capturing ground-dwelling arthropods, but extracting the arthropod specimens from the soil/litter samples requires considerable additional effort in the field, compared to servicing pitfall traps. When monitoring areas for which the reference libraries are well developed, arthropod species composition in soil samples can be determined through DNA metabarcoding of bulk samples, which obviates the need for individual specimen sorting [38]. Metabarcoding considerably reduces analytical costs per individual, compared to the specimen-based workflow employed in this study [34,39]. Any future large-scale monitoring efforts of Arctic arthropods, CBM-based or not, will likely need to employ metabarcoding for species identification in order to be economically feasible in the long term. A major drawback in metabarcoding is the lack of connection between DNA barcode sequences and the specimens from which they originate. DNA sequences linked to voucher specimens and corresponding valid taxon names produced by specimen-based workflows will remain critical for building reference libraries.

Rapid changes happening across the Arctic have led to growing acknowledgement of the importance of CBM in addressing the deficit of information required for decision-making at the local level. CBM is a key element of information exchange networks, such as the Conservation of Arctic Flora and Fauna circumpolar monitoring program (CAFF), the Exchange for Local Observations and Knowledge of Arctic (ELOKA), and the Sustainable Arctic Observing Network (SAON) [40]. In particular, there is considerable interest among researchers and local communities in using wildlife monitoring to detect change and to inform decisions [41]. Despite these developments, integration of CBM with scientific enquiry has been limited. A review of CBM articles from the past 33 years (1981–2014) indicated 244 peer-reviewed articles and 177 grey literature sources on CBM, with only 8.1% in the peer-reviewed literature and 18% in the grey literature representing the Arctic and Subarctic regions [40]. From the biodiversity research standpoint, there is a number of practical challenges to collecting essential biodiversity variables [42] such as the taxonomic impediment [43]. Applying DNA-based diagnostic framework would help overcome these shortfalls by providing a time- and cost-effective biodiversity information baseline for further enquiry into ecosystem processes.

## 5. Conclusions

Monitoring changes in the Arctic remains incomplete without data on arthropod communities. However, even baseline data for this speciose and ecologically important group are sparse and have limited taxonomic depth. A pilot effort to provide taxonomically comprehensive species-level biodiversity assessment of arthropods in the vicinity of Ikaluktutiak (Cambridge Bay), Nunavut, using DNA barcoding resulted in recording 1264 BINs (molecular proxy for species); Chao1 estimator was 1820 ± 73 BINs. Yellow pan traps alone captured 62% of the BINs, and their efficiency and ease of deployment makes them the preferred method for surveying and monitoring the terrestrial arctic arthropods. In order to sample ground-dwelling taxa, pan traps need to be complemented by other methods such as leaf litter and soil sampling.

Coupling community-based sample collection with high-throughput DNA barcoding has the potential to overcome or at least considerably alleviate some of the major obstacles to large-scale arthropod monitoring in the Arctic: travel cost of deploying research teams from southern institutions to conduct fieldwork, the difficulty of morphological identification of many arthropod species, and insufficient knowledge of taxonomy for many diverse taxa, such as soil mites. Data produced through such monitoring programs should be made available in real time to local stakeholders through online data portals, such as BOLD, providing required information about the state of biodiversity and facilitating data sharing and online collaboration with scientists, regulatory agencies, and between communities.

## Figures and Tables

**Table 1:** Types of collecting methods used and the habitat categories they targeted.

Habitat Category:	Active Collecting	Stationary Traps
**Aerial**	Sweep netting	Malaise trap, pan trap
**Vegetation**	Sweep netting, freehand collecting	
**Soil–surface**	Freehand collecting	Pitfall trap
**Soil–interstitial**	Sifting	
**Freshwater–aquatic**	Plankton net, D-net, aquatic hand-net	

**Table 2:** Summary of the studied material at the class and order level.

Class	Taxon	Specimens	BINs	Singletons	Doubletons	Chao1	Chao1 95% Cl Lower	Chao1 95% Cl Upper	Chao1 SD	% BINS Sampled	% Singletons	Upper-Lower Discrepancy
Insecta	Coleoptera	440	33	7	3	41.2	34.6	75.3	8.3	80.2%	21.2%	2.18
	Diptera	12,113	644	212	82	918.0	834.8	1037.6	51.1	70.2%	32.9%	1.24
	Ephemeroptera	78	3	0	0	not calculated						
	Hemiptera	312	19	10	2	43.9	24.1	141.9	24.2	43.3%	52.6%	5.90
	Hymenoptera	2402	255	91	39	361.1	316.8	437.2	29.8	70.6%	35.7%	1.38
	Lepidoptera	114	34	13	5	50.8	38.5	96.3	12.6	67.0%	38.2%	2.50
	Plecoptera	8	2	0	0							
	Thysanoptera	19	3	1	0	not calculated						
	Trichoptera	416	6	0	0	not calculated						
	Total Insecta	15,902	999	334	131	1424.8	1317.9	1567.4	63.1	70.1%	33.4%	1.19
Arachnida	Araneae	781	50	12	4	68.0	54.5	121.3	29.8	73.6%	24.0%	2.22
	Mesostigmata	74	20	7	4	not calculated						
	Sarcoptiformes	356	48	17	9	not calculated						
	Trombidiformes	415	74	32	13	not calculated						
	Unidentified Acari	40	20			not calculated						
	Total Acari	885	162	68	30	239.0	203.4	305.1	25.0	67.8%	42.0%	1.50
	Total Arachnida	1666	212	136	60	not calculated						
Collembola	Entomobryomorpha	106	26	8	1	not calculated						
	Poduromorpha	150	13	3	0	not calculated						
	Symphypleona	272	14	5	0	not calculated						
	Total Collembola	528	53	16	1	180.8	74.8	802.1	143.3	29.3%	30.2%	10.73
Total	Total Arthropoda	18,096	1264	430	166	1820.9	1695.4	1982.9	72.9	69.4%	34.0%	1.17

**Table 3:** Number of BINs recorded with each collecting method and the overlap (shared BINs) between methods.

BIN Count			BIN Overlap				
Method	BINs	Unique BINs	Aquatic	Malaise Trap	Pan Trap	Pitfall Trap	Sweep net
Aquatic	78	21					
Malaise Trap	413	112	40				
Pan Trap	787	359	38	262			
Pitfall Trap	155	12	10	50	121		
Terrestrial-aerial	455	125	42	186	246	82	
Terrestrial-soil	233	110	7	20	77	46	75

## Supplementary Materials

The following are available online at https://www.mdpi.com/2075-4450/11/1/46/s1. Table S1: GenBank accessions, BIN assignments, and detailed taxonomic data for the studied specimens. Table S2: Sequencing success statistics for the major arthropod taxa analyzed, and a comparison of the success rates between Malaise traps and pan traps.
